# Delineated domain of VP2 capsid protein in H-1 parvovirus that determines susceptibility to human cancer cells

**DOI:** 10.71150/jm.2601003

**Published:** 2026-05-27

**Authors:** Il-Rae Cho, Patcharporn Budluang, Yeon Ha Kim, Haan Park, Namuk Kim, Kon Ho Lee, Jin-Hyun Ahn, Ho Young Kang, Young-Hwa Chung

**Affiliations:** 1 Department of Cogno-Mechatronics Engineering, Optomechatronics Research Institute, Pusan National University, Busan 46241, Republic of Korea; 2 Department of Integrated Biological Science, Pusan National University, Busan 46241, Republic of Korea; 3 Department of Microbiology, School of Medicine, Gyeongsang National University, Jinju 52828, Republic of Korea; 4 Department of Microbiology, Sungkyunkwan University School of Medicine, Suwon 16419, Republic of Korea

**Keywords:** H-1 parvovirus, Kilham rat virus, tropism, chimeric virus, VP2 capsid protein, human cancer cell

## Abstract

Despite the application of H-1 parvovirus as an anticancer drug, the relationship between its specific tropism and oncolytic activity has been unknown. H-1 viral infection induced cytopathic effects in HeLa cells, whereas Kilham rat virus (KRV), similar to H-1 virus, did not. To explore which segments of the viral protein 2 (VP2) capsid protein in the H-1 virus determine susceptibility to human cancer cells, chimeric H-1 viruses with specific gene segments of H-1 VP2 were constructed. Delineation of the VP2 capsid protein revealed a minimum domain (K208–L435 in the H-1 VP2 protein) to determine infectivity in human cancer cells; however, this domain was not sufficient to maintain infectivity. To solve this problem, further construction of chimeric H-1 viruses illustrated the necessity of segments covering both M1-N87 and D104-P206 in the H-1 VP2 protein, based on chimeric H-1 viruses designated as YCH44, YCH45, and YCH46. Both the variable region 4b (VR4b) domains from KRV VP2 and VR8 from H-1 VP2 were required for the same purpose, based on chimeric H-1 viruses designated as YCH-HK8, YCH16, YCH17, YCH18, and YCH19. We confirmed that chimeric viruses carrying these segments infected human lung adenocarcinoma A549 and pancreatic cancer Panc-1 cells, whereas the parental KRV did not. Taken together, these findings indicate that specific domains of the H-1 virus VP2 capsid protein determine infectivity toward human cancer cells.

## Introduction

Oncolytic viruses have been utilized as an alternative strategy for cancer treatment because of an increase in the number of cancer cells that are resistant to chemotherapeutic drugs or radiation therapy (Beljanski and Hiscott, 2012; Hemminki et al., 2020; Tamura et al., 2013; Ylösmäki and Cerullo, 2020). Oncolytic viruses more favorably infect and replicate in cancer cells than in normal cells, and also induce the infiltration of T cells recognizing oncolytic viruses and tumor antigens (Kaufman et al., 2015). Oncolytic viruses are classified as immunotherapeutic drugs. Oncolytic viruses currently employed in clinical cancer therapy include reovirus, vaccinia virus, and α-herpesvirus (Conry et al., 2018).

H-1 parvovirus is a small, single-stranded DNA virus with a genome of approximately 5.1 kb that encodes two nonstructural proteins (NS1 and NS2) and two capsid proteins (VP1 and VP2). VP1 is a minor capsid protein composed of a unique N-terminal extension fused to the VP2 capsid domain. The major capsid protein, VP2, is essential for capsid assembly (Vihinen-Ranta et al., 2002). NS1 protein has multiple functions such as helicase, ATPase, DNA-nicking, and sequence-speciﬁc DNA binding activities essential for DNA replication (Christensen and Tattersall, 2002; Rhode, 1989). NS2 protein is also involved in DNA amplication (Ruiz et al., 2011). The X-ray crystal structure of the H-1 VP2 protein has been compared with those of the minute virus of mouse (MVMp) and canine parvovirus (CPV). Ten VRs within the H-1 VP2 protein—designated VR0, VR1, VR2, VR3, VR4a, VR4b, VR5, VR6, VR7, and VR8—have been identified through comparisons with MVMp and CPV (Halder et al., 2013). Although rodent cells constitute the natural hosts of H-1 parvovirus, the virus is also capable of infecting transformed human cells (Vollmers and Tattersall, 2013). In permissive cells, H-1 virus replicates during the S phase of the cell cycle and proceeds through a lytic infection, ultimately inducing apoptosis and lysosome-mediated cell death (Di Piazza et al., 2007; Rayet et al., 1998). Owing to these properties, H-1 parvovirus has been explored as an anticancer agent for the treatment of glioblastoma and pancreatic cancer, not only in animal models but also in clinical settings (Fakhiri et al., 2019; Hajda et al., 2021).

Kilham rat virus (KRV), a member of the Parvoviridae family like H-1 parvovirus, replicates efficiently in its natural rodent hosts (Besselsen et al., 1995). Notably, KRV is known to induce autoimmune type I diabetes in rats (Chung et al., 2000; Guberski et al., 1991; Zipris et al., 2003). Despite its close similarity to KRV, H-1 parvovirus does not elicit autoimmune diabetes in the same rodent model (Zipris et al., 2003).

In this study, we observed that H-1 virus produced pronounced cytopathic effects in HeLa cells, whereas KRV did not. This prompted us to investigate the specific domain of the H-1 VP2 capsid protein responsible for determining susceptibility in human cancer cells. To address this question, we generated a series of chimeric H-1 viruses incorporating various H-1 VP2 domains. Our findings demonstrate that the K208–L435 region of the H-1 VP2 protein is a key determinant for infectivity in human cancer cells. However, this region alone was insufficient to propagate the chimeric H-1 virus, which also required additional segments corresponding to the M1–N87 and D104–P206 domains of the H-1 VP2 protein. Furthermore, we identified two variable regions-VR4b from KRV VP2 and VR8 from H-1 VP2-as essential for generating infectious H-1 chimeric viruses.

## Materials and Methods

### Cell cultures and transfection

Rat-1, Normal rat kidney (NRK), A549 (human lung adenocarcinoma), and Panc-1 (human pancreatic ductal carcinoma) cells were obtained from ATCC (USA), and HeLa cells were purchased from the Korean Cell Line Bank (Korea). All cell lines were maintained in Dulbecco’s modified Eagle’s medium (DMEM; Gibco, USA) supplemented with 10% fetal bovine serum and 1% penicillin–streptomycin (Gibco), and cultured at 37°C in a humidified atmosphere containing 5% CO_2_. The cells were cultured at a density of 5 × 10^5^ cells in a 60 mm culture plate for 24 h before transfection. Cells with 70–80% confluence were then transfected with 2 μg of DNA using Lipofectamine 2000 (Invitrogen, USA).

### Immunoblotting

The harvested cells were lysed in lysis buffer [150 mM NaCl, 1% NP-40, 50 mM Tris-HCl (pH 7.5)] supplemented with 0.1 mM Na_2_VO_3_, 1 mM NaF, and a protease inhibitor cocktail (Sigma-Aldrich, USA). Proteins from the lysates were separated by 10% SDS–polyacrylamide gel electrophoresis and subsequently transferred onto nitrocellulose membranes. Primary antibodies were applied at a 1:1000 dilution, followed by horseradish peroxidase–conjugated secondary antibodies at a 1:2000 dilution in 5% nonfat dry milk. After the final washes, protein bands were visualized using enhanced chemiluminescence and imaged with an ImageQuant LAS 4000 Mini system (GE Healthcare, Japan).

### Sequencing of KRV genomic DNA

NRK cells were infected with KRV and recovered 24 h after infection, and KRV replicative DNA was isolated as previously described (Gunther and May, 1976). The KRV NS1 and NS2 genes were cloned into the pCR2.1-Topo vector (Invitrogen), based on a published H-1 viral genomic sequence. The pBluescript II SK(+) vector, which includes the KRV replicative form DNA spanning the EcoRI and HpaI sites, was used to sequence the KRV VP1 and VP2 genes. Sequencing was conducted by Macrogen (Korea), and the sequenced KRV NS1, NS2, VP1, and VP2 genes were deposited in GenBank (accession nos. KM999994–999997).

### Construction of chimeric H-1 viruses

Based on VP2 gene sequence of H-1 (JX505432) and KRV (KM999997) deposited in GenBank, several VP2 recombinant genes were designed and synthesized by Bioneer (Korea). Synthesized double stranded recombinant VP2 genes were cloned into the pBHA vector (Bioneer). The infectious H-1 virus DNA clone (pSR19) (Rhode and Paradiso, 1983) which was then modified by the insertion of NotI sites at the front of the HpaI site, was used as the backbone vector for the construction of chimeric H-1 viruses (Fig. 2B). To insert the recombinant VP2 genes into the modified pSR19 vector, HindIII and HpaI restriction enzyme sites were used and new chimeric vectors were constructed. The new recombinant vectors were confirmed by digestion with HindIII and NotI (Fig. 2C). For the production of chimeric viruses, each chimeric vector was transfected into Rat-1 cells, which were harvested 5 days post-transfection (dpt). The cell supernatants were inoculated into fresh HeLa cells for amplification of chimeric viruses, and the viruses were confirmed by VP2 DNA sequencing after viral DNA isolation. Viral titers were measured as the 50% tissue culture infective dose per ml (TCID_50_/ml).

### Production of polyclonal H-1 antibodies

NRK cell monolayers were infected with wild-type H-1 virus until a cytopathic effect became evident (approximately 60% cell lysis). Cells were collected by low-speed centrifugation at 500 × g for 15 min at 10°C. Viral particles were released from the pelleted cells through three rapid freeze–thaw cycles and subsequently purified by two rounds of sucrose discontinuous gradient centrifugation (first gradient: 5–50%; second gradient: 10–40%) at 72,000 × g for 12 h using an SW28 rotor (Beckman Coulter, USA) at 10°C. The purity and integrity of the viral preparations were confirmed by 15% SDS–polyacrylamide gel electrophoresis followed by Coomassie Blue staining. Purified and inactivated H-1 viruses (200 μg/injection), formulated with appropriate adjuvants, were administered subcutaneously to rabbits at 2-week intervals. The resulting sera were collected to obtain a polyclonal H-1 antibodies, which recognize the H-1 VP1 as well as VP2 capsid protein.

### Modeling of VP2 capsid protein from KRV and H-1 virus

A structural model of the KRV VP2 protein (accession no. KM999997) was generated using SWISS-MODEL, with the H-1 parvovirus VP2 structure (PDB ID: 4G0R) (Halder et al., 2013) serving as the template. The template was selected based on its high amino acid sequence identity to KRV VP2 (79%), identified using the Basic Local Alignment Search Tool (BLAST), and was retrieved from the Protein Data Bank at the highest available resolution. Molecular visualization and structural analyses were conducted using the UCSF Chimera software package (Pettersen et al., 2004).

### Statistical analysis

Data are presented as the Mean ± standard deviation (SD) and were subjected to one-way ANOVA followed by Tukey’s test for comparisons between groups. A *p*-value < 0.05 was considered significant.

## Results

### Comparison of genomic DNA and amino-acid sequences between KRV and H-1 virus

**Fig. 1.**
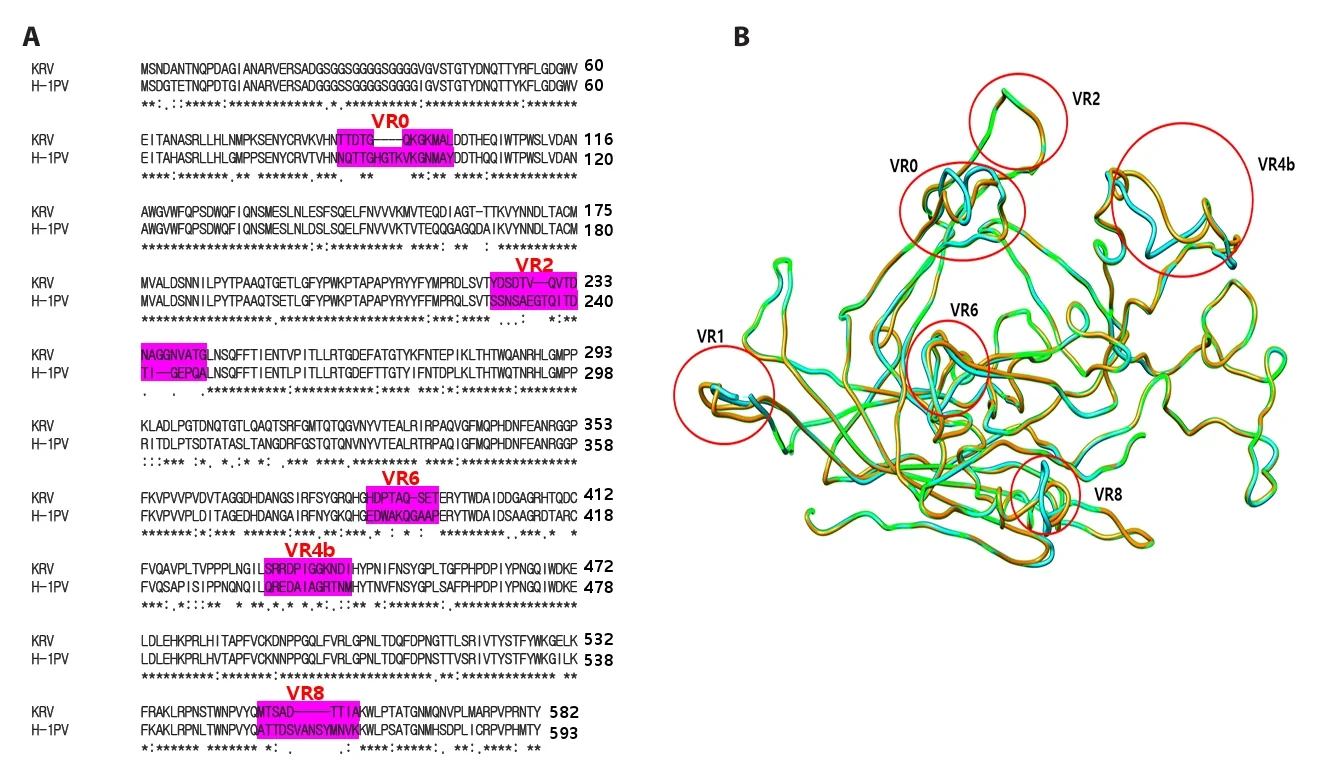
Amino acid sequence and three-dimensional modeling of VP2 capsid protein between KRV and H-1 parvovirus. (A) Alignment of VP2 amino-acid sequence between KRV and H-1 virus. (B) Superposition of coil representations of VP2 of KRV and H-1 virus. Cyan and yellow color indicate VP2 of H-1 virus and KRV, respectively. Green yellow indicates superposition of VP2 protein between them.

Although KRV and H-1 are known to be similar (Zipris et al., 2003), their infectivity patterns have not yet been characterized in detail. To investigate this, we first sequenced the genomic DNA of KRV obtained from the ATCC and compared it with the H-1 viral genome. As summarized in Table 1, the NS1 and NS2 proteins of H-1 and KRV exhibit over 99% nucleotide and amino acid sequence homology. In contrast, the VP1 genes share 86.2% nucleotide sequence homology and 78.8% amino acid sequence homology, while the VP2 genes display 83.3% nucleotide and 73.4% amino acid sequence homology. The results suggest that NS1 and NS2 are critical for the shared elements of the KRV and H-1 viral life cycles. Additionally, the roughly 20% difference in the amino acid sequences of the capsid proteins VP1 and VP2 appears to confer distinct properties to each virus, contributing to differences in their individual life cycles. As 10 VRs have been reported in the H-1 VP2 capsid protein in comparison with other parvoviruses, such as MVMp and CPV (Halder et al., 2013), the VRs between the H-1 virus and KRV were found at almost identical positions (Fig. 1A). Herein, we designated more variable parts of the amino acid sequence of VP2 capsid proteins, such as VR0, VR2, VR6, VR4b, and VR8, based on their primary and three-dimensional structure (Fig. 1B). Except for VR2, the other VRs in the VP2 protein exhibited different three-dimensional structures in the H-1 virus and KRV (Fig. 1B).

### Minimum segment of H-1 VP2 capsid protein to warrant infection of human cancer cells

**Fig. 2.**
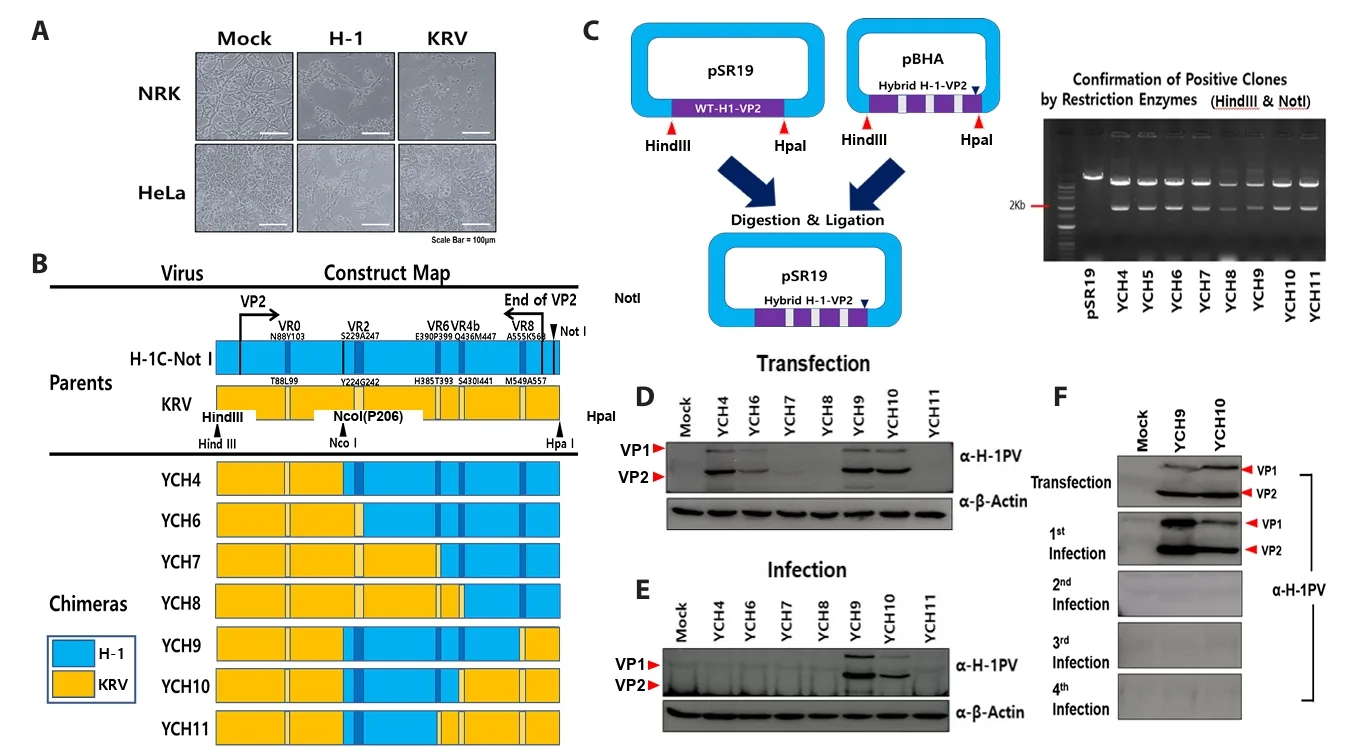
Identification of a core H-1 VP2 gene segment responsible for HeLa cell infectivity. (A) HeLa and NRK cells were infected with KRV and H-1 parvoviruses at MOI of 1. Cell morphology was examined under light microscopy 72 h post-infection. Scale bars = 100 µm. Magnification = ×200. (B) Schematic diagrams of chimeric KRV vectors containing a core gene segment of the H-1 VP2 protein. (C) To construct chimeric viruses, VP2 capsid proteins were designed, synthesized, and cloned into the pBHA vector based on the VP2 sequences of H-1 and KRV. The pSR19 vector, carrying the full-length infectious H-1 genome, was modified by replacing its VP2 region with the recombinant VP2 sequences through HindIII and HpaI digestion. Successful incorporation of the chimeric VP2 segments was confirmed by digestion with HindIII and NotI, the latter recognizing a site introduced into the chimeric VP2 genes. (D–F) Rat-1 cells were transfected with the recombinant vectors, and cell lysates were collected five days post-transfection. Expression of the recombinant VP2 proteins was analyzed by immunoblotting using polyclonal H-1 virus antibodies that can also recognize VP1 proteins. Supernatants from the transfected cells were then used to infect HeLa cells for three days, and the generation of chimeric viruses was confirmed by immunoblotting. Subsequently, supernatants were harvested repeatedly and used to inoculate HeLa cells for four successive passages, with propagation of the chimeric viruses monitored by immunoblotting using polyclonal H-1 virus antibodies.

Previous studies have documented subtle differences in the capsid proteins of CPV and feline panleukopenia virus (FPV), as well as between lymphotropic and fibrotropic MVM, which dictate species and tissue tropism, respectively (Kontou et al., 2005; Nam et al., 2006; Parker and Parrish, 1997; Truyen and Parrish, 1992; Truyen et al., 1994). Likewise, the amino acid sequences of the H-1 and KRV capsid proteins differ by approximately 20%, suggesting that these viruses may exhibit distinct tropism patterns in various cell lines. Consistent with their natural rodent hosts, NRK cells were susceptible to both H-1 and KRV infections (Fig. 2A). Interestingly, while human cervical HeLa cancer cells were permissive to H-1 infection, they were resistant to KRV (Fig. 2A).

To identify the segment of the H-1 virus VP2 protein responsible for infecting human cancer cells, several chimeric VP2 genes (YCH4, YCH6–YCH11) were designed (Fig. 2B), synthesized by Bioneer, and used to replace the corresponding wild-type H-1 gene segment in the pSR19 vector (Fig. 2C), as previously described (Halder et al., 2013; Paglino and Tattersall, 2011). Rat-1 cells were transfected with recombinant pSR19 vectors containing chimeric VP2 genes (YCH4, YCH6–YCH11) that are double stranded DNA. At 5 dpt, the cells were harvested, and lysed via three cycles of freeze-thawing. Immunoblot analysis detected H-1 VP1 and VP2 capsid proteins only in YCH4/pSR19, YCH6/pSR19, YCH9/pSR19, and YCH10/pSR19-transfected cells (Fig. 2D). Supernatants from these lysates were then inoculated into HeLa cells to produce infectious chimeric viruses. Three days post-inoculation, HeLa cells were harvested, and cell lysates were analyzed by immunoblotting using polyclonal H-1 antibodies. Infectious chimeric H-1 viruses were obtained from YCH9/pSR19 and YCH10/pSR19-transfected lysates, whereas no infectious virus was produced from YCH4/pSR19 and YCH6/pSR19-transfected cells (Fig. 2E). These results indicate that chimeric viral assembly occurs properly with YCH9/pSR19 and YCH10/pSR19, allowing the production of infectious viruses. Furthermore, the blue segment in YCH10, corresponding to K208–L435 of the H-1 VP2 protein, appears to be the minimal domain required for generating infectious chimeric parvoviruses. However, infectious viruses from both YCH9/pSR19 and YCH10/pSR19 lysates were not maintained during a second round of infection, as they lost infectivity (Fig. 2F). This suggests that additional segments of the H-1 VP2 gene are necessary to propagate the chimeric viruses derived from YCH9/pSR19 and YCH10/pSR19.

### Necessity of N-terminal H-1 VP2 gene segments for construction of chimeric H-1 virus with sustained infectivity

**Fig. 3.**
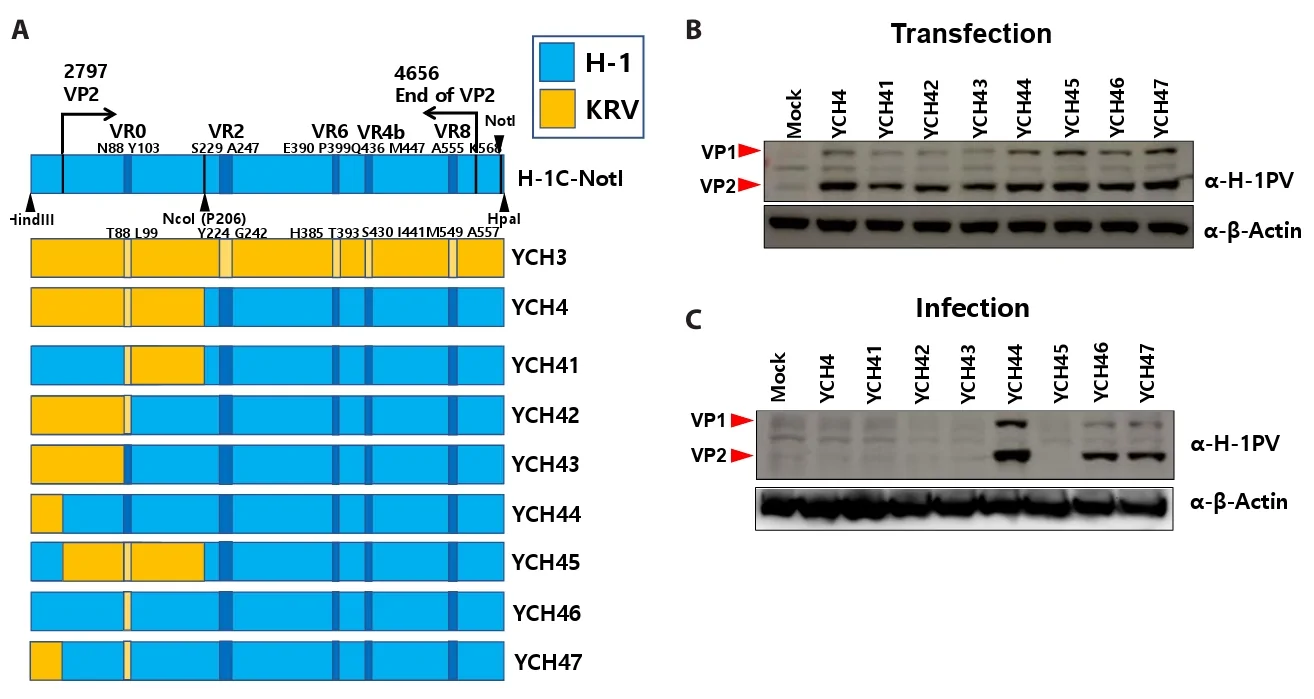
N-terminal gene segments of H-1 VP2 protein are required for the construction of infectious chimeric viruses. (A) Schematic representation of chimeric H-1 vectors containing different N-terminal gene segments of the H-1 VP2 protein. YCH3 indicates a gene segment from the complete KRV genome digested with HindIII and HpaI, encompassing the entire KRV VP2 capsid protein (from M1 to the terminal Y582 residue). (B, C) Rat-1 cells were transfected with the recombinant vectors, and cell lysates were collected five days post-transfection. Expression of the recombinant VP2 proteins was analyzed by immunoblotting using polyclonal H-1 virus antibodies that can also recognize VP1 proteins. Following three cycles of freeze-thawing of the infected HeLa cells, supernatants from the transfected cell lysates were used to infect HeLa cells for three days, and successful construction of the chimeric viruses was confirmed by immunoblotting using polyclonal H-1 virus antibodies.

As no infectious virus was generated from the YCH4/pSR19 vector (Fig. 2E), gene segments from the N-terminal domain of the H-1 VP2 protein were deemed necessary for the YCH4 construct. Accordingly, several chimeric VP2 genes were designed and synthesized in the pSR19 vector (YCH41, YCH42, YCH43, YCH44, YCH45, YCH46, and YCH47) (Fig. 3A). All recombinant vectors were transfected into Rat-1 cells (Fig. 3B), and expression of VP1 and VP2 proteins was confirmed in the transfected cell lysates (Fig. 3B). Infectious chimeric parvoviruses were obtained from YCH44/pSR19-, YCH46/pSR19-, and YCH47/pSR19-transfected lysates, whereas YCH41/pSR19-, YCH42/pSR19-, YCH43/pSR19-, and YCH45/pSR19-transfected cells did not produce infectious virus (Fig. 3C). These results indicate that the H-1 VP2 gene segments spanning M1–N87 and D104–P206 are critical for the successful construction of infectious chimeric parvoviruses.

**Fig. 4.**
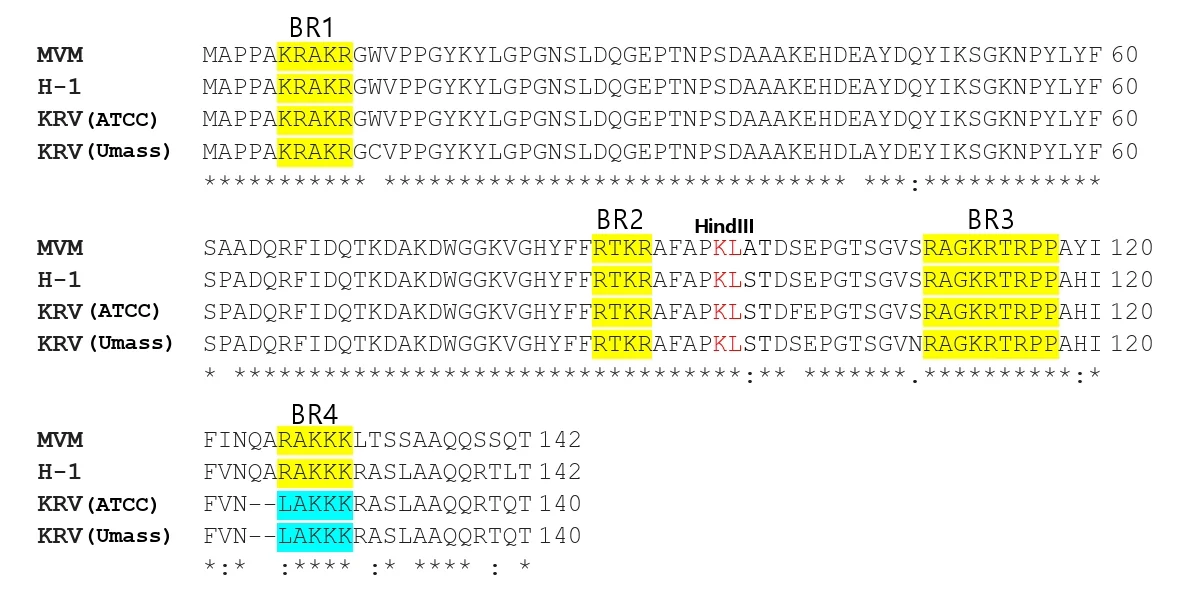
VP1 unique regions of KRVs and H-1 parvovirus responsible for nuclear localization. Alignment of amino acid sequences of VP1 unique regions of MVM (AAA67114.1), KRV (ATCC VR-1790; KM999997.1), KRV (University of Massachusetts; AAB38328.1), and H-1 (AFR44451.1).

Additionally, a nuclear localization sequence, which plays a critical role in MVM viral replication (Lombardo et al., 2002), was identified in the VP1 unique region of both H-1 and KRV. As shown in Fig. 4, the amino acid sequences of basic region 1 (BR1), BR2, and BR3 were identical between H-1 and KRV (ATCC or University of Massachusetts isolate), whereas BR4 exhibited minor differences. Notably, a chimeric virus from the YCH46 vector, derived from the H-1 VP1 unique region, was successfully produced, similar to YCH47, which originated from the KRV VP1 unique region (Fig. 3A and 3C). These results indicate that the VP1 unique region of H-1 is not required for generating infectious chimeric viruses. Furthermore, comparison of infectious chimeras from YCH44 and YCH47 vectors revealed that VR0 (N88–Y103) of H-1 VP2 is also dispensable for the production of infectious chimeric virus (Fig. 3A and 3C).

### Necessity of C-terminal H-1 VP2 gene segments for the construction of chimeric H-1 viruses with sustained infectivity

**Fig. 5.**
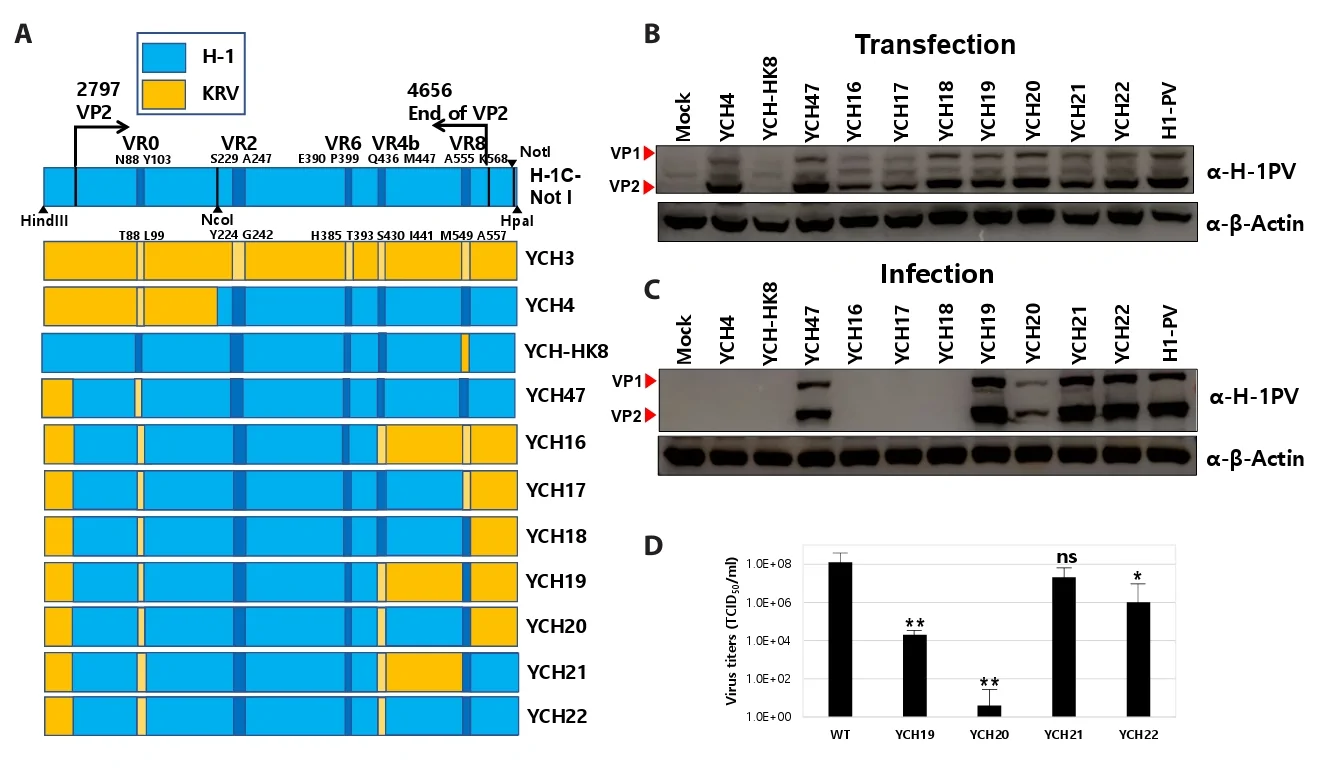
C-terminal gene segments of H-1 VP2 capsid protein are required for the construction of infectious chimeric viruses. (A) Schematic representation of chimeric H-1 vectors containing various C-terminal gene segments of the H-1 VP2 protein. The YCH-HK8 vector was generated by introducing the KRV VR8 domain into the H-1 VP2 gene. (B–D) Rat-1 cells were transfected with the recombinant vectors, and cell lysates were collected at 5 dpt. Expression of the recombinant VP2 proteins was analyzed by immunoblotting using polyclonal H-1 virus antibodies that can also recognize VP1 proteins. Following three cycles of freeze-thawing of the infected HeLa cells, supernatants from the transfected cell lysates were used to infect HeLa cells for three days, and successful construction of the chimeric viruses was confirmed by immunoblotting. The viral titers of the infectious chimeric viruses (YCH19, YCH20, YCH21, and YCH22) were then determined by TCID_50_/ml after four consecutive passages in HeLa cells. Virus tiers of the chimeric viruses were compared to that of wild type H-1 virus (*p<0.05, **p<0.01, ns: not significant).

Building on previous results showing that the M1–N87 and D104–P206 segments of H-1 VP2 are required for generating infectious chimeric viruses with sustained infectivity, we incorporated these segments into new chimeric VP2 vectors. Accordingly, several chimeric vectors were designed and synthesized (YCH-HK8, YCH16–YCH22) to include various combinations of C-terminal H-1 VP2 gene segments (Fig. 5A). All recombinant vectors were transfected into Rat-1 cells, and expression of VP1 and VP2 proteins was confirmed in cell lysates, with the exception of YCH-HK8 (Fig. 5B). Infectious chimeric parvoviruses were successfully generated from YCH19/pSR19-, YCH20/pSR19-, YCH21/pSR19-, and YCH22/pSR19-transfected lysates, whereas YCH16/pSR19, YCH17/pSR19, and YCH18/pSR19 did not produce infectious virus (Fig. 5C). Based on these results, we drew several conclusions. First, comparison of HeLa cell infectivity among YCH-HK8, YCH16, and YCH19 vectors indicated that VR8 (A555–K568) of H-1 is essential for the production of infectious chimeric virus. Second, comparison between YCH18 and YCH20 vectors revealed that VR4b from KRV, rather than H-1, is required for generating infectious chimeras. Third, regarding consecutive amplification, the YCH20/pSR19-derived chimeric virus exhibited the lowest viral titers, while YCH19/pSR19 had the third lowest (Fig. 5D). These results suggest that achieving high-titer chimeric viruses requires H-1 gene segments H448–Q554 and K569–Y593, or K569–Y593 alone. Furthermore, after amplification of YCH19, YCH20, YCH21, and YCH22 chimeric viruses, the VP2 capsid protein gene sequences were confirmed from the isolated viral DNA genomes (Data S1).

### Chimeric viruses enable infection of other human cancer cells as seen in HeLa cells

**Fig. 6.**
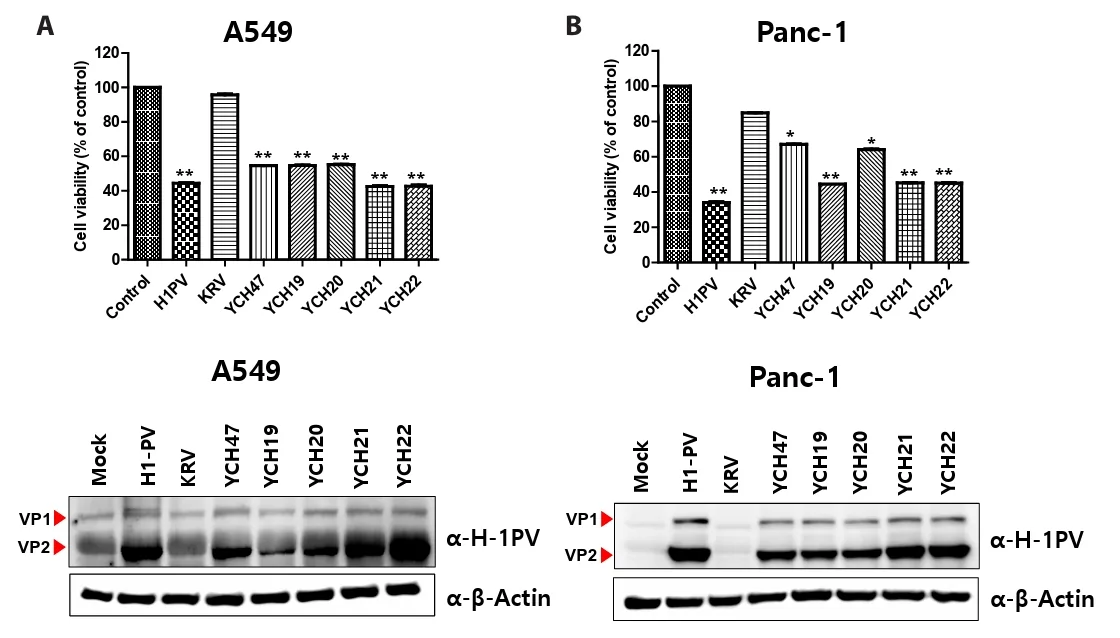
The infectious chimeric viruses reproduce H-1-specific tropism in human A549 and Panc-1 cancer cells. (A, B) Human A549 lung and Panc-1 cancer cells were infected with the chimeric viruses (YCH47, YCH19, YCH20, YCH21, and YCH22) as well as the parental KRV and H-1 parvoviruses at MOI of 1. Cell viability was assessed 72 h post-infection using the MTT assay. Data represent the mean of triplicate wells, with error bars indicating SD. Cell viability in cultures infected with parental H-1 virus or the chimeric viruses was compared to that in KRV-infected cells (*p<0.05, **p<0.01). Cell lysates were prepared for detection of viral propagation by immunoblotting using polyclonal H-1 virus antibodies.

Since the chimeric viruses YCH47 and YCH19–YCH22, which contain a partial H-1 VP2 capsid domain, induced cytolysis in HeLa cells, we extended our analysis to other human cell lines. Accordingly, these viruses were introduced into human lung adenocarcinoma A549 and pancreatic cancer Panc-1 cells, alongside the parent KRV and H-1 viruses. Infected cells were monitored and quantified using the MTT assay for up to 72 h post-infection (Fig. 6A and 6B). We discovered that A549 and Panc-1 cells exhibited cytolysis when infected with H-1 and chimeric viruses designated YCH47, YCH19, YCH20, YCH21, and YCH22, but were resistant to KRV infection (Fig. 6A and 6B). The results suggest that the chimeric H-1 viruses could mimic H-1 specific tropism by showing the same infectivity toward human cancer cells.

## Discussion

In this study, recombinant H-1 viruses with infectivity toward human cancer cells were developed using the pSR19 vector containing the full H-1 genome by introducing chimeric VP2 genes derived from H-1 and KRV. These results suggest that specific domains within the H-1 VP2 gene play an important role in infectivity toward human cancer cells. Consistent with this notion, a previous study (Paglino and Tattersall, 2011) reported that insertion of the LuIII VP2 gene into the complete genome of MVM, which normally replicates poorly in human cancer cells, conferred robust replication in human cancer cells, comparable to that of wild-type LuIII parvovirus, indicating a critical role for VP2 in determining infectivity. Nevertheless, it is reasonable to pursue the identification of recombinant viruses in which specific H-1 VP2 domains are incorporated into a KRV backbone to confer infectivity toward human cancer cells. Therefore, these approaches will be undertaken in future studies.

Herein, it is not well understood how infectious virus particles are generated following transfection with the pSR19 vector, which contains the entire H-1 viral genome within M13 backbone (Rhode and Paradiso, 1983). Since the size of the pSR19 vector exceeds 10 kb, it is generally understood that H-1 viral particles cannot encapsidate more than 10 kb of double-stranded DNA. Thus, during transfection with recombinant pSR19 vectors, viral particles lacking the H-1 viral genome or containing recombinant H-1 single-stranded DNA (~5 kb) may be generated. Based on evidence reported to date (Halder et al., 2013; Mietzsch et al., 2019; Paglino and Tattersall, 2011; Parrish, 2010), we attempt to propose a plausible mechanism as follows. The H-1 viral genome contains two promoters, promoter 4 (P4) and promoter 38 (P38). Transcription from P4 produces NS1 and NS2 mRNAs, leading to the expression of the corresponding proteins, whereas transcription from P38 generates VP1 and VP2 mRNAs and their proteins. We propose that the NS1 and NS2 proteins, which are involved in viral DNA replication, utilize the vector-derived DNA as a template for replication. Subsequently, the capsid proteins (VP1 and VP2) package the replicated H-1 DNA, resulting in the formation of infectious viral particles. However, during the assembly of recombinant VP2 proteins into virions, there is a possibility that the recombinant DNA fails to be packaged into the virion, resulting in the formation of empty particles. Alternatively, the recombinant VP2 protein may fail to assemble into virions. Finally, it is also conceivable that the recombinant VP2 protein is incomplete and is degraded immediately after synthesis. Meanwhile, in the case of recombinant viruses derived from the YCH9/pSR19 and YCH10/pSR19 vectors, we observed that although infectious virus particles were generated during first round of infection, they failed to replicate upon secondary infection. Several explanations are possible for this phenomenon; in addition to possibility of gene instability, we aim to describe the results more accurately by considering multiple potential mechanisms. Considering the viral life cycle, these possibilities include defects in host cell entry, endosomal trafficking, nuclear transport, uncoating, viral DNA replication, or capsid assembly. Accordingly, we herein introduced consecutive infections of the chimeric H-1 viruses to identify chimeric H-1 viruses capable of sustained infectivity as part of a VP2 recombination strategy.

The polyclonal H-1 antibodies prepared in this study recognize not only the H-1 viral VP2 capsid protein but also the KRV VP2 and the recombinant VP2 capsid protein. To explain this result, we quote a previous report presenting the three-dimensional structural model of the H-1 virus (Halder et al., 2013). The H-1 virion is composed of approximately 10 copies of the VP1 protein and 50 copies of the VP2 capsid protein (Halder et al., 2013). In addition to VR0, VR2, VR4b, VR6, and VR8, the remaining variable regions-VR1, VR3, VR5, VR4a, and VR7-also contribute to the formation of the icosahedral structure and are exposed on the virion surface (Halder et al., 2013). Since these VR regions (VR1, VR3, VR5, VR4a, and VR7) have amino acid sequences that are nearly identical or highly similar between the H-1 virus and KRV, antibodies that recognize the H-1 virus are likely to also recognize KRV VP2 as well as the recombinant VP2 effectively.

We observed that the H-1 virus induced cytopathic effects in HeLa, A549, and Panc-1 cells, whereas KRV did not, despite their ~80% sequence similarity. Construction of the chimeric virus YCH10 suggested that a specific domain of the H-1 VP2 protein (K208–L435) is critical for infectivity in human cancer cells, although YCH10 did not retain infectivity. Subsequent generation of chimeric viruses with sustained infectivity indicated that additional domains, M1–N87 and D104–P206, in H-1 VP2 are also required. In contrast, the variable region VR0 appeared dispensable for the production of stable chimeric viruses, despite its distinct three-dimensional structure in H-1 and KRV. Further analyses revealed that both the VR4b domain from KRV and the VR8 domain from H-1 VP2 are absolutely necessary for viral propagation. For example, YCH18 did not produce infectious recombinant virus despite sharing the same H-1-derived VR8 as YCH19–YCH22. However, it should be noted that in YCH18, VR4b is derived from the H-1 virus, whereas in YCH19–YCH22, VR4b is derived from KRV. YCH16 also failed to produce infectious recombinant virus despite containing a KRV-derived VR4b, likely because it does not possess a VR8 segment derived from the H-1 virus. In contrast, for YCH47, all gene segments at the C-terminus are identical to those of the wild-type H-1 virus, which could reasonably explain the successful generation of infectious recombinant virus. Therefore, under the combined condition that VR4b is derived from KRV and VR8 is derived from H-1 virus, the origin of the gene segments located between VR4b and VR8, as well as those from VR8 to the C-terminal end of VP2, does not appear to substantially influence the maintenance of infectivity of the recombinant viruses, regardless of whether these segments are derived from KRV or H-1 virus. However, based on chimeric virus titers observed for YCH21 and YCH22, we suggest that either the combined H448–Q554 and K569–Y593 domains (as in YCH22) or the single K569–Y593 domain (as in YCH21) is preferential for chimeric viral propagation. For example, although YCH-HK8 vector contains both H-1 virus-derived gene segments (H448–Q554 and K569–Y593), it fails to produce infectious recombinant virus. This observation may be attributable to the fact that VR8 in this construct is derived from KRV. Moving forward, we aim to elucidate the detailed mechanisms by which these specific VP2 domains in chimeric viruses (YCH19, YCH20, YCH21, and YCH22) govern H-1 virus tropism.

We observed that the wild-type H-1 virus and recombinant viruses carrying specific H-1 VP2 domains were able to infect not only the cervical cancer cell line HeLa, but also the lung cancer cell line A549 and the pancreatic cancer cell line Panc-1, resulting in viral replication. Because we did not examine the infectivity or replication of the H-1 virus or the specific recombinant H-1 viruses in normal human cells or non-transformed human cells, it is insufficient to conclude that these viruses infect cancer cells in a cancer-specific manner. However, previous studies (Geiss et al., 2017; Paglino and Tattersall, 2011) have shown that H-1 virus exhibits markedly lower viral replication in normal fibroblast cell lines and non-transformed osteoblast cell lines compared with transformed cell lines. Therefore, we cautiously suggest that the H-1 virus and specific recombinant H-1 viruses are capable of infecting and replicating in human cancer cells.

Our results align with previous studies. For instance, one study showed that MVM subtypes (lymphotropic and fibrotropic) are determined by only two amino acid residues in the MVM capsid protein (Ball-Goodrich and Tattersall, 1992). Similarly, investigations of CPV and FPV revealed that specific sequences within the VP1 and VP2 structural protein genes dictate host range, despite the high overall sequence similarity between these viruses (Parker and Parrish, 1997; Truyen and Parrish, 1992; Truyen et al., 1994). Building on studies of MVM that identified VP2 residues involved in sialic acid binding, residues I367 and H373 on the H-1 VP2 capsid protein have been suggested as critical for host cell interaction (Allaume et al., 2012; López-Bueno et al., 2006). Here, we identify V362 and H370 on the KRV VP2 capsid protein as the residues corresponding to I367 and H373 in H-1, potentially mediating sialic acid binding. Given the similar biochemical properties of isoleucine (I367) in H-1 and valine (V362) in KRV, we propose that additional host cell molecules, beyond sialic acid, may contribute to viral entry and thereby influence H-1- or KRV-specific tropism. This hypothesis is supported by evidence that parvovirus entry can involve multiple receptors, such as the transferrin receptor for CPV and FPV (Park et al., 2005; Parker et al., 2001) and heparan sulfate, αvβ5 integrin, or growth factor receptors for adeno-associated viruses (AAV) (Kashiwakura et al., 2005; Summerford and Samulski, 1998; Summerford et al., 1999), even though sialic acid acts as a common attachment factor for H-1, MVM, AAV1, AAV4, CPV, and FPV (López-Bueno et al., 2006). These findings suggest that multiple host molecules may participate in parvovirus entry. Therefore, the roles of specific domains in the chimeric H-1 VP2 protein during early viral life cycle events, including host cell molecule-mediated viral entry, will be examined in future studies. Furthermore, when examining the specific tropism of these recombinant viruses, we will not attribute the observed effects solely to viral entry. Instead, we will investigate viral trafficking from the endosome to the nucleus, uncoating, viral DNA replication, capsid assembly, and packaging of viral DNA to provide a more comprehensive explanation of these phenomena, which could provide insights into the distinct tropism patterns exhibited by different rodent parvoviruses.

## Figures and Tables

**Table 1.** Comparison between H-1PV and KRV genome

Genes	Matched nucleotides	Nucleotide homology (%)	Matched amino acids	Amino acid homology (%)
NS1	2014/2019	99.8	668/672	99.4
NS2	564/567	99.8	186/188	98.9
VP1	1836/2205	86.2	594/734	78.8
VP2	1414/1779	83.3	443/592	73.4

## Supplementary Information

Supplementary Data S1. Recombinant H-1 viruses propagated in HeLa cells were purified by high-speed centrifugation using a discontinuous sucrose gradient (10–40%) at 72,000 × g for 12 h, and the viral particles were collected. Viral DNAs of the recombinant H-1 viruses were subsequently isolated and purified from the recovered viral suspension using a viral genome isolation kit (Qiagen). Based on the VP2 sequence information provided by GenBank (KM999997), primers were designed at 500-bp intervals. DNA sequencing was performed by Bioneer Co., Ltd.

Supplementary Material 1. Author note
