# Supplementary material for: Delineated domain of VP2 capsid protein in H-1 parvovirus that determines susceptibility to human cancer cells
Source: J Microbiol. 2026 May 27;64(5):e2601003. doi: 10.71150/jm.2601003 (PMC13577013; doi:10.71150/jm.2601003)
Supplement: Supplementary file 1 [file jm-2601003-Supplementary-Data-S1.pdf]

Data S1. Recombinant H-1 viruses propagated in HeLa cells were purified by high-speed centrifugation using a discontinuous sucrose gradient (10–40%) at 72,000 × g for 12 h, and the viral particles were collected. Viral DNAs of the recombinant H-1 viruses were subsequently isolated and purified from the recovered viral suspension using a viral genome isolation kit (Qiagen). Based on the VP2 sequence information provided by GenBank (KM999997), primers were designed at 500-bp intervals. DNA sequencing was performed by Bioneer Co., Ltd.

Chimeric H-1- YCH19 DNA sequence

AAGCTTTCTACTGACTTTGAACCTGGCACTTCTGGTGTGAGCAGAGCTGGTAAACGAACTA
AACCACCTGCTCACATTTTTGTAAATCTAGCTAAAAAAAAACGCGCATCTCTTGCTGCTCA
GCAGAGGACTCAGACTATGAGTGATGGCACCGAAACAAACCAACCAGACACTGGAATCG
CTAATGCTAGAGTTGAGCGATCAGCTGACGGAGGTGGAAGCTCTGGGGGTGGGGGCTCTG
GCGGGGGTGGGATTGGTGTTTCTACTGGGACTTATGATAATCAAACGACTTATAAGTTTTTG
GGAGATGGATGGGTAGAAATAACTGCACATGCTTCTAGACTTTTGCACTTGGGAATGCCTC
CTTCAGAAAACTACTGCCGCGTCACCGTTCACAATACAACAGACACAGGACAAAAAGGGA
AAATGGCTCTTGATGACACACATCAACAAATTTGGACACCATGGAGCTTGGTAGATGCTAA
TGCTTGGGGAGTTTGGTTCCAACCAAGTGACTGGCAGTTCATTCAAAACAGCATGGAATC
GCTGAATCTTGACTCATTGAGCCAAGAACTATTTAATGTAGTAGTCAAAACAGTCACTGAA
CAACAAGGAGCTGGCCAAGATGCCATTAAAGTCTATAATAATGACTTGACGGCCTGTATGA
TGGTTGCTCTGGATAGTAACAACATACTGCCTTACACACCTGCAGCTCAAACATCAGAAAC
ACTTGGTTTCTACCCATGGAAACCAACCGCACCAGCTCCTTACAGATACTACTTTTTCATGC
CTAGACAACTCAGTGTAACCTCTAGCAACTCTGCTGAAGGAACTCAAATCACAGACACCA
TTGGAGAGCCACAGGCACTAAACTCTCAATTTTTTACTATTGAGAACACCTTGCCTATTACT
CTCCTGCGCACAGGTGATGAGTTTACAACTGGCACCTACATCTTTAACACTGACCCACTTA
AACTTACTCACACATGGCAAACCAACAGACACTTGGGCATGCCTCCAAGAATAACTGACC
TACCAACATCAGATACAGCAACAGCATCACTAACTGCAAATGGAGACAGATTTGGATCAAC
ACAAACACAGAATGTGAACTATGTCACAGAGGCTTTGCGCACCAGGCCTGCTCAGATTGG
CTTCATGCAACCTCATGACAACTTTGAAGCAAACAGAGGTGGCCCATTTAAGGTTCCAGTG
GTACCGCTAGACATAACAGCTGGCGAGGACCATGATGCAAACGGAGCCATACGATTTAACT
ATGGCAAACAACATGGCGAAGATTGGGCCAAACAAGGAGCAGCACCAGAAAGGTACACA
TGGGATGCAATTGATAGTGCAGCTGGGAGGGACACAGCTAGATGCTTTGTACAAAGTGCA
CCAATATCTATTCCACCAAACCAAAACCAGATCTTGTCAAGAAGAGACCCAATAGGTGGTA
AGAATGACATTCATTATCCAAACATTTTTAACAGCTACGGTCCACTAACTGGATTTCCTCAT
CCAGACCCTATCTACCCGAATGGGCAGATCTGGGACAAAGAACTTGACCTGGAGCACAAA
CCTAGACTGCATATCACTGCACCATTTGTCTGTAAAGACAACCCGCCAGGTCAACTCTTTG

TTCGCTTGGGACCTAACCTAACAGACCAATTTGATCCAAACGGCACAACTCTCTCTAGAAT
TGTTACATATAGCACCTTCTACTGGAAAGGTGAACTGAAATTCAGAGCCAAACTCAGACCA
AACTCTACATGGAACCCAGTGTACCAAGCAACCACAGACTCTGTTGCCAATTCTTACATGA
ATGTTAAGAAATGGCTACCAACTGCAACAGGTAACATGCAGAATGTGCCACTTATGGCCAG
ACCTGTACCTCGCAACACATACTAACCAACCAACTATGCTTCTCTGTCTGCTTCACATAATA
CTTAGACTAACTAGACTGCAACATAGAAATATACACTTAATAATAGATTATAGAAATAACATA
ATATGCGGCCGCTACTGGTTAAC

Chimeric H-1- YCH20 DNA sequence

AAGCTTTCTACTGACTTTGAACCTGGCACTTCTGGTGTGAGCAGAGCTGGTAAACGAACTA
AACCACCTGCTCACATTTTTGTAAATCTAGCTAAAAAAAAACGCGCATCTCTTGCTGCTCA
GCAGAGGACTCAGACTATGAGTGATGGCACCGAAACAAACCAACCAGACACTGGAATCG
CTAATGCTAGAGTTGAGCGATCAGCTGACGGAGGTGGAAGCTCTGGGGGTGGGGGCTCTG
GCGGGGGTGGGATTGGTGTTTCTACTGGGACTTATGATAATCAAACGACTTATAAGTTTTTG
GGAGATGGATGGGTAGAAATAACTGCACATGCTTCTAGACTTTTGCACTTGGGAATGCCTC
CTTCAGAAAACTACTGCCGCGTCACCGTTCACAATACAACAGACACAGGACAAAAAGGGA
AAATGGCTCTTGATGACACACATCAACAAATTTGGACACCATGGAGCTTGGTAGATGCTAA
TGCTTGGGGAGTTTGGTTCCAACCAAGTGACTGGCAGTTCATTCAAAACAGCATGGAATC
GCTGAATCTTGACTCATTGAGCCAAGAACTATTTAATGTAGTAGTCAAAACAGTCACTGAA
CAACAAGGAGCTGGCCAAGATGCCATTAAAGTCTATAATAATGACTTGACGGCCTGTATGA
TGGTTGCTCTGGATAGTAACAACATACTGCCTTACACACCTGCAGCTCAAACATCAGAAAC
ACTTGGTTTCTACCCATGGAAACCAACCGCACCAGCTCCTTACAGATACTACTTTTTCATGC
CTAGACAACTCAGTGTAACCTCTAGCAACTCTGCTGAAGGAACTCAAATCACAGACACCA
TTGGAGAGCCACAGGCACTAAACTCTCAATTTTTTACTATTGAGAACACCTTGCCTATTACT
CTCCTGCGCACAGGTGATGAGTTTACAACTGGCACCTACATCTTTAACACTGACCCACTTA
AACTTACTCACACATGGCAAACCAACAGACACTTGGGCATGCCTCCAAGAATAACTGACC
TACCAACATCAGATACAGCAACAGCATCACTAACTGCAAATGGAGACAGATTTGGATCAAC
ACAAACACAGAATGTGAACTATGTCACAGAGGCTTTGCGCACCAGGCCTGCTCAGATTGG
CTTCATGCAACCTCATGACAACTTTGAAGCAAACAGAGGTGGCCCATTTAAGGTTCCAGTG
GTACCGCTAGACATAACAGCTGGCGAGGACCATGATGCAAACGGAGCCATACGATTTAACT
ATGGCAAACAACATGGCGAAGATTGGGCCAAACAAGGAGCAGCACCAGAAAGGTACACA
TGGGATGCAATTGATAGTGCAGCTGGGAGGGACACAGCTAGATGCTTTGTACAAAGTGCA
CCAATATCTATTCCACCAAACCAAAACCAGATCTTGTCAAGAAGAGACCCAATAGGTGGTA
AGAATGACATTCATTATACTAATGTTTTTAACAGCTATGGTCCACTTAGTGCATTTCCTCATC

CAGATCCCATTTATCCAAATGGACAAATTTGGGACAAAGAATTGGACCTGGAACACAAACC
TAGACTACACGTAACTGCACCATTTGTTTGTAAAAACAACCCACCAGGTCAACTATTTGTT
CGCTTGGGGCCTAATCTGACTGACCAATTTGACCCAAACAGCACAACTGTTTCTCGCATTG
TTACATATAGCACTTTTTACTGGAAGGGTATTTTGAAATTCAAAGCCAAACTAAGACCAAAT
CTGACCTGGAATCCTGTATACCAAGCAACCACAGACTCTGTTGCCAATTCTTACATGAATGT
TAAGAAATGGCTACCAACTGCAACAGGTAACATGCAGAATGTGCCACTTATGGCCAGACCT
GTACCTCGCAACACATACTAACCAACCAACTATGCTTCTCTGTCTGCTTCACATAATACTTA
GACTAACTAGACTGCAACATAGAAATATACACTTAATAATAGATTATAGAAATAACATAATAT
GCGGCCGCTACTGGTTAAC

Chimeric H-1- YCH21 DNA sequence

AAGCTTTCTACTGACTTTGAACCTGGCACTTCTGGTGTGAGCAGAGCTGGTAAACGAACTA
AACCACCTGCTCACATTTTTGTAAATCTAGCTAAAAAAAAACGCGCATCTCTTGCTGCTCA
GCAGAGGACTCAGACTATGAGTGATGGCACCGAAACAAACCAACCAGACACTGGAATCG
CTAATGCTAGAGTTGAGCGATCAGCTGACGGAGGTGGAAGCTCTGGGGGTGGGGGCTCTG
GCGGGGGTGGGATTGGTGTTTCTACTGGGACTTATGATAATCAAACGACTTATAAGTTTTTG
GGAGATGGATGGGTAGAAATAACTGCACATGCTTCTAGACTTTTGCACTTGGGAATGCCTC
CTTCAGAAAACTACTGCCGCGTCACCGTTCACAATACAACAGACACAGGACAAAAAGGGA
AAATGGCTCTTGATGACACACATCAACAAATTTGGACACCATGGAGCTTGGTAGATGCTAA
TGCTTGGGGAGTTTGGTTCCAACCAAGTGACTGGCAGTTCATTCAAAACAGCATGGAATC
GCTGAATCTTGACTCATTGAGCCAAGAACTATTTAATGTAGTAGTCAAAACAGTCACTGAA
CAACAAGGAGCTGGCCAAGATGCCATTAAAGTCTATAATAATGACTTGACGGCCTGTATGA
TGGTTGCTCTGGATAGTAACAACATACTGCCTTACACACCTGCAGCTCAAACATCAGAAAC
ACTTGGTTTCTACCCATGGAAACCAACCGCACCAGCTCCTTACAGATACTACTTTTTCATGC
CTAGACAACTCAGTGTAACCTCTAGCAACTCTGCTGAAGGAACTCAAATCACAGACACCA
TTGGAGAGCCACAGGCACTAAACTCTCAATTTTTTACTATTGAGAACACCTTGCCTATTACT
CTCCTGCGCACAGGTGATGAGTTTACAACTGGCACCTACATCTTTAACACTGACCCACTTA
AACTTACTCACACATGGCAAACCAACAGACACTTGGGCATGCCTCCAAGAATAACTGACC
TACCAACATCAGATACAGCAACAGCATCACTAACTGCAAATGGAGACAGATTTGGATCAAC
ACAAACACAGAATGTGAACTATGTCACAGAGGCTTTGCGCACCAGGCCTGCTCAGATTGG
CTTCATGCAACCTCATGACAACTTTGAAGCAAACAGAGGTGGCCCATTTAAGGTTCCAGTG
GTACCGCTAGACATAACAGCTGGCGAGGACCATGATGCAAACGGAGCCATACGATTTAACT
ATGGCAAACAACATGGCGAAGATTGGGCCAAACAAGGAGCAGCACCAGAAAGGTACACA
TGGGATGCAATTGATAGTGCAGCTGGGAGGGACACAGCTAGATGCTTTGTACAAAGTGCA

CCAATATCTATTCCACCAAACCAAAACCAGATCTTGTCAAGAAGAGACCCAATAGGTGGTA
AGAATGACATTCATTATCCAAACATTTTTAACAGCTACGGTCCACTAACTGGATTTCCTCAT
CCAGACCCTATCTACCCGAATGGGCAGATCTGGGACAAAGAACTTGACCTGGAGCACAAA
CCTAGACTGCATATCACTGCACCATTTGTCTGTAAAGACAACCCGCCAGGTCAACTCTTTG
TTCGCTTGGGACCTAACCTAACAGACCAATTTGATCCAAACGGCACAACTCTCTCTAGAAT
TGTTACATATAGCACCTTCTACTGGAAAGGTGAACTGAAATTCAGAGCCAAACTCAGACCA
AACTCTACATGGAACCCAGTGTACCAAGCAACCACAGACTCTGTTGCCAATTCTTACATGA
ATGTTAAGAAATGGCTCCCATCTGCAACTGGCAACATGCACTCTGATCCATTGATTTGTAGA
CCTGTGCCTCACATGACATACTAACCAACCAACTATGTTTCTCTGTTTGCTTCACATAATACT
TAAACTAACTAGACTACAACATAAAAATATACACTTAATAATAGATTATAAAAATAACATAAT
ATGCGGCCGCTATTGGTTAAC

Chimeric H-1- YCH22 DNA sequence

AAGCTTTCTACTGACTTTGAACCTGGCACTTCTGGTGTGAGCAGAGCTGGTAAACGAACTA
AACCACCTGCTCACATTTTTGTAAATCTAGCTAAAAAAAAACGCGCATCTCTTGCTGCTCA
GCAGAGGACTCAGACTATGAGTGATGGCACCGAAACAAACCAACCAGACACTGGAATCG
CTAATGCTAGAGTTGAGCGATCAGCTGACGGAGGTGGAAGCTCTGGGGGTGGGGGCTCTG
GCGGGGGTGGGATTGGTGTTTCTACTGGGACTTATGATAATCAAACGACTTATAAGTTTTTG
GGAGATGGATGGGTAGAAATAACTGCACATGCTTCTAGACTTTTGCACTTGGGAATGCCTC
CTTCAGAAAACTACTGCCGCGTCACCGTTCACAATACAACAGACACAGGACAAAAAGGGA
AAATGGCTCTTGATGACACACATCAACAAATTTGGACACCATGGAGCTTGGTAGATGCTAA
TGCTTGGGGAGTTTGGTTCCAACCAAGTGACTGGCAGTTCATTCAAAACAGCATGGAATC
GCTGAATCTTGACTCATTGAGCCAAGAACTATTTAATGTAGTAGTCAAAACAGTCACTGAA
CAACAAGGAGCTGGCCAAGATGCCATTAAAGTCTATAATAATGACTTGACGGCCTGTATGA
TGGTTGCTCTGGATAGTAACAACATACTGCCTTACACACCTGCAGCTCAAACATCAGAAAC
ACTTGGTTTCTACCCATGGAAACCAACCGCACCAGCTCCTTACAGATACTACTTTTTCATGC
CTAGACAACTCAGTGTAACCTCTAGCAACTCTGCTGAAGGAACTCAAATCACAGACACCA
TTGGAGAGCCACAGGCACTAAACTCTCAATTTTTTACTATTGAGAACACCTTGCCTATTACT
CTCCTGCGCACAGGTGATGAGTTTACAACTGGCACCTACATCTTTAACACTGACCCACTTA
AACTTACTCACACATGGCAAACCAACAGACACTTGGGCATGCCTCCAAGAATAACTGACC
TACCAACATCAGATACAGCAACAGCATCACTAACTGCAAATGGAGACAGATTTGGATCAAC
ACAAACACAGAATGTGAACTATGTCACAGAGGCTTTGCGCACCAGGCCTGCTCAGATTGG
CTTCATGCAACCTCATGACAACTTTGAAGCAAACAGAGGTGGCCCATTTAAGGTTCCAGTG
GTACCGCTAGACATAACAGCTGGCGAGGACCATGATGCAAACGGAGCCATACGATTTAACT

ATGGCAAACAACATGGCGAAGATTGGGCCAAACAAGGAGCAGCACCAGAAAGGTACACA
TGGGATGCAATTGATAGTGCAGCTGGGAGGGACACAGCTAGATGCTTTGTACAAAGTGCA
CCAATATCTATTCCACCAAACCAAAACCAGATCTTGTCAAGAAGAGACCCAATAGGTGGTA
AGAATGACATTCATTATACTAATGTTTTTAACAGCTATGGTCCACTTAGTGCATTTCCTCATC
CAGATCCCATTTATCCAAATGGACAAATTTGGGACAAAGAATTGGACCTGGAACACAAACC
TAGACTACACGTAACTGCACCATTTGTTTGTAAAAACAACCCACCAGGTCAACTATTTGTT
CGCTTGGGGCCTAATCTGACTGACCAATTTGACCCAAACAGCACAACTGTTTCTCGCATTG
TTACATATAGCACTTTTTACTGGAAGGGTATTTTGAAATTCAAAGCCAAACTAAGACCAAAT
CTGACCTGGAATCCTGTATACCAAGCAACCACAGACTCTGTTGCCAATTCTTACATGAATGT
TAAGAAATGGCTCCCATCTGCAACTGGCAACATGCACTCTGATCCATTGATTTGTAGACCTG
TGCCTCACATGACATACTAACCAACCAACTATGTTTCTCTGTTTGCTTCACATAATACTTAAA
CTAACTAGACTACAACATAAAAATATACACTTAATAATAGATTATAAAAATAACATAATATGC
GGCCGCTATTGGTTAAC
