# Supplementary material for: Delineated domain of VP2 capsid protein in H-1 parvovirus that determines susceptibility to human cancer cells
Source: J Microbiol. 2026 May 27;64(5):e2601003. doi: 10.71150/jm.2601003 (PMC13577013; doi:10.71150/jm.2601003)
Supplement: Supplementary file 2 [file jm-2601003-Supplementary-Material-1.pdf]

Journal of
Microbiology

Author note

Journal of Microbiology [Epub ahead of print]
https://doi.org/10.71150/jm.2601003
pISSN 1225-8873 • eISSN 1976-3794

Author note: Delineated domain of VP2 capsid protein in H-1 parvovirus that determines susceptibility to human cancer cells

Il-Rae Cho[1,†], Patcharporn Budluang[1,†,#], Yeon Ha Kim[2], Haan Park[2], Namuk Kim[2], Kon Ho Lee[3], Jin-Hyun Ahn[4], Ho Young Kang[2,*], Young-Hwa Chung[1,*]

[1]Department of Cogno-Mechatronics Engineering, Optomechatronics Research Institute, Pusan National University, Busan 46241, Republic of Korea
[2]Department of Integrated Biological Science, Pusan National University, Busan 46241, Republic of Korea
[3]Department of Microbiology, School of Medicine, Gyeongsang National University, Jinju 52828, Republic of Korea
[4]Department of Microbiology, Sungkyunkwan University School of Medicine, Suwon, 16419, Republic of Korea
[#]Present address: Department of Pharmacology, Faculty of Medicine, Chiang Mai University, Chiang Mai 50200, Thailand

***Correspondence**
Ho Young Kang
hoykang@pusan.ac.kr

Young-Hwa Chung
younghc@pusan.ac.kr

[†]These authors contributed equally to this work.

The article entitled "VP2 capsid domain of the H-1 parvovirus determines susceptibility of human cancer cells to H-1 viral infection" was published in Cancer Gene Therapy 22:271–277 (2015). Subsequently, we conducted additional experiments to further delineate the VP2 capsid domain of H-1 parvovirus and assess its role in determining the susceptibility of human cancer cells, using chimeric constructs designated CH4 and CH5. However, we later discovered that both CH4 and CH5 constructs had been contaminated with wild-type H-1 virus. We voluntarily reported this issue, which led to the withdrawal of the article in 2021. In the present study, we redesigned and synthesized new chimeric constructs and confirmed their integrity by DNA sequencing to avoid cross-contamination. Our findings first demonstrate that the K208-L435 region of the H-1 VP2 protein is a key determinant for infectivity in human cancer cells. However, this region alone was insufficient to propagate the chimeric H-1 virus, which also required additional segments corresponding to the M1-N87 and D104-P206 domains of the H-1 VP2 protein. Furthermore, we identified two variable regions-VR4b from KRV VP2 and VR8 from H-1 VP2-as essential for generating infectious H-1 chimeric viruses.

References

Cho IR, Kaowinn S, Song J, Kim S, Koh SS, et al. 2015. RETRACTED ARTICLE: VP2 capsid domain of the H-1 parvovirus determines susceptibility of human cancer cells to H-1 viral infection. *Cancer Gene Ther.* 22: 271–277.
Cho IR, Kaowinn S, Song J, Kim S, Koh SS, et al. 2021. Retraction Note: VP2 capsid domain of the H-1 parvovirus determines susceptibility of human cancer cells to H-1 viral infection. *Cancer Gene Ther.* 28: 350.

© The Author(s), under exclusive licence to Microbiological Society of Korea 2025
